# Screening and Assessment of Potential Plant Growth-promoting Bacteria Associated with *Allium cepa* Linn.

**DOI:** 10.1264/jsme2.ME19147

**Published:** 2020-03-06

**Authors:** Brian Estuardo Samayoa, Fo-Ting Shen, Wei-An Lai, Wen-Ching Chen

**Affiliations:** 1 International Master Program of Agriculture, National Chung-Hsing University, Taichung, Taiwan 40227; 2 Department of Soil and Environmental Science, National Chung-Hsing University, Taichung, Taiwan 40227; 3 Innovation and Development Center of Sustainable Agriculture (IDCSA), National Chung Hsing University, Taichung, Taiwan 40227; 4 International Bachelor Program of Agribusiness, National Chung-Hsing University, Taichung, Taiwan 40227

**Keywords:** *Allium cepa* Linn., indole acetic acid, nitrogen fixation, ACC deaminase, plant growth promotion

## Abstract

Plant growth-promoting bacteria (PGPB) are beneficial microbes that increase plant growth and yield. However, limited information is currently available on PGPB in onion (*Allium cepa* Linn.). The aims of the present study were to isolate and identify PGPB in onion and examine the effects of isolated PGPB on germination and growth during the vegetative stage in onion, pak choy (*Brassica chinensis*), and sweet pepper (*Capsicum annuum*). Twenty-three strains of PGPB were isolated from the roots, bulbs, and rhizosphere soil of onion. All isolated bacterial strains showed one or more PGP traits, including indole acetic acid production, phosphate solubilization ability, and 1-aminocyclopropane-1-carboxylate deaminase and nitrogenase activities; most of these traits were derived from *Bacillus* sp., *Microbacterium* sp., and *Pseudomonas* sp. Eight bacteria that exhibited strong abilities to produce indole acetic acid were selected for a Petri dish trial, soil pot test, and vermiculate pot test. The Petri dish trial showed that strains ORE8 and ORTB2 simultaneously increased radicle and hypocotyl lengths in onion, but inhibited growth in sweet pepper after 7 d. The soil pot experiment on onion revealed that strains ORE5, ORE8, and ORTB2 strongly promoted growth during the vegetative stage with only a half dose of chemical fertilizer. The present results indicate that ORE8 (*Bacillus megaterium*) and ORTB2 (*Pantoea* sp.) are the most promising biofertilizers of onion and may simultaneously inhibit the seedling growth of other plants.

Modern crop production involves protecting the environment, maintaining soil fertility, and controlling pests effectively while achieving reasonable yield. This task is more challenging in Taiwan, located in a subtropical region, in which small and fragmented cultivated lands and high temperature and humidity all contribute to serious competition between pests and crops. The use of chemical fertilizers and pesticides has always been the main approach to promote agricultural production in Taiwan. However, the long-term use of chemical fertilizers and pesticides has affected the original environment, deteriorated soil fertility, generated highly resistant pests, and raised public concerns about the environment and health.

In recent years, plant growth-promoting bacteria (PGPB) have attracted increasing attention because they promote the growth of crops such as maize, rice, and sugarcane (Almaghrabi *et al.*, 2014; Prittesh *et al.*, 2017; Santos *et al.*, 2018). The application of one or more PGPB strains to fields enhanced the growth of crops while reducing the need for chemical fertilizers (Adesemoye *et al.*, 2009), thereby representing a beneficial approach to more sustainable agricultural practices. Although previous studies reported the positive and negative effects of PGPB on onion (*Allium cepa* Linn.) (Jha *et al.*, 2005; Čolo *et al.*, 2014), few PGPB have been isolated from onion even though Taiwan ranks third in the world for onion yield hectare^–1^ (Fatih, 2018). Due to the low absorption and penetration abilities of the onion’s root system as well as the high potential for weed invasion during planting, onion is a suitable candidate for the application of PGPB.

Some bacteria suppress plant growth by overexpressing PGP traits, particularly the production of indole acetic acid (IAA). The seedling growth of radish was previously shown to be suppressed by high IAA-producing *Enterobacter* sp. I-3 (Park *et al.*, 2015). *E. taylorae* was also reported to inhibit the growth of weeds through the production of high levels of IAA (Sarwar and Kremer, 1995). Auxin herbicides mimic the structure of naturally occurring IAA and its derivatives and have become some of the major synthetic herbicides that selectively control broad-leaved weeds because monocots (such as onion) perceive or respond differently to exogenous auxins than dicots (Carlile, 2006; McSteen, 2010; El-Ghit, 2016).

Many bacteria have been shown to simultaneously function as PGPB and biopesticides, such as *Pseudomonas fluorescence* and *Bradyrhizobium elkanii* (Vijayan *et al.*, 2013; Trognitz *et al.*, 2016; David *et al.*, 2018). These effects, combined with the findings that some isolated PGPB did not effectively increase plant growth (Vejan *et al.*, 2016), suggest the importance of screening potential PGPB in seedling growth, pot, or field experiments because PGP genes also commonly exist in non-PGPB Proteobacteria (Bruto *et al.*, 2014).

In the present study, we isolated possible biofertilizers associated with onion that exhibited strong PGP characteristics. We examined their effects on onion and two other crops at different plant growth stages. Microbial flora with the potential for biofertilization were also screened. These strains were then used as bioinoculants to examine their effects on plant growth.

## Materials and Methods

### Isolation of A. cepa-associated bacteria

Onion plants (*A. cepa* Linn., cultivar: Golden No. 5) were collected from a farmer’s plantation in Taichung County, Taiwan. To isolate onion root endophytes, the roots were rinsed with distilled water to remove debris and then sterilized with 75% alcohol for 15‍ ‍min, followed by immersion in 6% sodium hypochlorite for another 15‍ ‍min. Roots were washed with sterilized water after the sterilization process. Samples were ground as described previously (Lai *et al.*, 2008). The extract was serially diluted 10-fold over a range between 10^–2^ and 10^–8^, and a 100-μL cell suspension was plated onto nutrient agar (NA; HiMedia) plates. The same procedure was used to isolate strains from bulbs. However, the method of Matthysse and McMahan (1998) was used to isolate rhizosphere and rhizoplane bacteria with slight modifications. Approximately 3.5 cm of the root tip was washed in 9 mL sterile reverse osmosis (RO) water twice, and the two washes were combined as loosely bound bacteria. The washed roots were placed into glass tubes containing 9 mL sterile water and 10 glass beads with a diameter of 2.5 mm and were then vigorously vortexed for 1‍ ‍min. A cell suspension of tightly bound bacteria was obtained. The two fractions were plated on NA plates. The bacteria isolated from the onion bulbs, endophytic root, and root surface (both loosely bound and tightly bound to the root surface) were designated as OBE, ORE, ORLB, and ORTB, respectively, in Table 2.

### Phosphate solubilization ability

Bacterial isolates were screened for phosphate solubilization properties using Pikovskaya medium (containing KCl, 0.2 g L^–1^; MgSO_4_·7H_2_O, 0.1 g L^–1^; glucose, 10 g L^–1^; yeast extract, 0.5 g L^–1^; FeSO_4_·7H_2_O, 0.002 g L^–1^; MnSO_4_·H_2_O, 0.002 g L^–1^; [NH_4_]_2_ SO_4_, 0.5 g L^–1^; Ca_3_[PO_4_]_2_, 5 g L^–1^). Bacterial strains were inserted into the middle of the medium plates using sterilized tips. Plates were incubated at 30°C for 7‍ ‍d to monitor the positive or negative development of a clear halo around the colonies.

### IAA-like compound production ability

To test IAA-like compound production abilities, each bacterial strain was inoculated into test tubes containing 5 mL NB (HiMedia) and incubated at 30°C for 24 h on a rotating shaker (180 rpm). A 20-μL amount of the culture was transferred into 5‍-‍mL test tubes containing NB with 500‍ ‍μg mL^–1^ tryptophan and shaken at 180 rpm for 42 h. The cell density of cultures was measured at 600 nm. To measure the IAA-like compound in the culture, 1.5 mL of the culture from 500‍ ‍μg mL^–1^ tryptophan containing NB was centrifuged at 5,500×*g* at 4°C for 10‍ ‍min. A 1-mL amount of the appropriately diluted sample was placed into 4 mL of Salkowski reagent. The absorbance of samples was measured at 535 nm after an incubation at room temperature for 20‍ ‍min in the dark (Patten and Glick, 2002).

### 1-Aminocyclopropane-1-carboxylate (ACC) deaminase activity

Experiments to measure ACC deaminase in strains were conducted as described previously (Penrose and Glick, 2003). Each bacterial strain was inoculated into 5-mL test tubes containing NB (HiMedia) and shaken at 180 rpm for 24 h. A 30-μL amount of the bacterial culture was transferred into 5-mL test tubes containing Dworkin-Foster (DF) salts minimal medium with 3‍ ‍mM ACC and shaken at 180 rpm for 2 d. The absorbance of the culture was measured at 600 nm. A 1.5-mL bacterial culture was centrifuged at 8,000×*g* at 4°C for 10‍ ‍min. Bacteria cell pellets were collected and washed with 1 mL of 0.1 M Tris-HCl (pH 7.6) at 4°C and washed cell pellets were re-suspended in 600‍ ‍μL of 0.1 M Tris-HCl (pH 8.5). A 30-μL amount of toluene was added to the cell suspension and vortexed for 30 s. The enzymatic reaction was initiated by adding 20‍ ‍μL ACC to 200‍ ‍μL of toluenized cells after a pre-incubation at 30°C for 3‍ ‍min. After a 15-min reaction, 1 mL of 0.56 M HCl was added. The mixture was collected and centrifuged at 10,000×*g* at room temperature for 5‍ ‍min. A 1-mL amount of the supernatant with 800‍ ‍μL of 0.56 M HCl was vortexed. A 300-mL amount of 2,4-dinitrophenylhydrazine reagent was added and incubated at 30°C for 30‍ ‍min. Two milliliters of 2 N NaOH was added to each test tube and absorbance was recorded at 540 nm. The known concentration range of 0 to 1.0 mmol α-ketobutyrate was used as a standard.

### Nitrogen fixation ability

Nitrogen fixation was evaluated using the acetylene reduction assay (Rennie, 1981). After growing on NA (HiMedia) plates for 3‍ ‍d, bacteria were introduced into test tubes with nitrogen-free semi-solid medium. After an incubation at 30°C for 4‍ ‍d, the screw caps of the test tubes were replaced with serum stoppers. A 2-mL amount of acetylene gas was injected into the test tubes using a 5‍-‍mL syringe and left to stand at 30°C for 24 h. To quantify ethylene, a 0.5-mL amount of gas from the tube was injected into a gas chromatograph with a flame ionization detector (Model 163; HITACHI). The conditions of the analysis were carrier gas, nitrogen; flow rate, 35 mL h^–1^; temperature of the flame ionization detector, 110°C; column (1 m×2 mm steel column packed with a Porapak-T 80–100 mesh); temperature, 80°C.

### Identification of bacterial strains

Bacterial isolates were identified by targeting 16S rDNA (Willems and Collins, 1993). The primer pair 27F 5′-AGAGTTTGATCCTGGCTCAG-3′; 1492R 5′-GGTTACCTTGTTACGACTT-3′ was used to amplify a 1,500-bp region. PCR amplification involved a 50-μL reaction mixture consisting of 50‍ ‍ng genomic DNA, 20 pmol of each primer, 1.25 units Taq DNA polymerase, 200‍ ‍μM of each dNTP, and 1× PCR buffer. PCR was performed for 32 cycles in a Mycycler (Bio-Rad) with initial denaturation at 95°C for 3‍ ‍min, cyclic denaturation at 94°C for 30‍ ‍s, annealing at 58°C for 30‍ ‍s, and extension at 72°C for 2‍ ‍min with a final extension of 7‍ ‍min at 72°C. The amplicon of each reaction was analyzed by 1% agarose gel staining with ethidium bromide and visualized under UV light with a gel documentation system. Amplicons were sent to Genomics BioSci & Tech for sequencing.

16S rRNA gene sequences were identified by a BLAST search on Sep 15, 2019, and were deposited to NCBI database (MN685245–MN685267). Closely related type strains were obtained from NCBI GenBank via the EzTaxon server (http://www.eztaxon.org). The 16S rRNA gene sequences obtained were analyzed using the sequence alignment Blast program from NCBI (https://blast.ncbi.nlm.nih.gov/Blast.cgi) and then aligned with the sequences of closely related strains using CLUSTAL_X 1.83 (Thompson *et al.*, 1997). The software package MEGA 6 (Tamura *et al.*, 2013) was used to construct phylogenetic trees with the neighbor-joining method (Saitou and Nei, 1987). A bootstrap analysis (Felsenstein, 1985) was performed according to the algorithm of Kimura’s two-parameter model (Kimura, 1980) based on 1,000 resamplings (Felsenstein, 1993).

### Petri dish introduction of selected bacterial strains in three crops

Among the 23 strains isolated from the onion plant parts, 8 showing high IAA production abilities were introduced into three vegetable crops—onion, pak choy (*Brassica chinensis*), and sweet pepper (*Capsicum annuum*)—to examine their effects on the germination of seeds and elongation of radicle and hypocotyl lengths. Strains were grown on NA plates for 3‍ ‍d and then suspended in RO water. A 2-mL amount of the cell suspension at A_600_=0.1 was added to filter paper (AdvantecToyo) in a Petri dish. Seeds were surface-sterilized in 1% sodium hypochlorite for 15‍ ‍min. Plates were then incubated at 30°C for 7 d. We used five replicates for each strain. The hypocotyl and radicle lengths of three crops were measured after 7‍ ‍d of culture.

### Soil pot trial on selected bacterial strains in onion

Among the eight bacterial strains in the Petri dish test, five were selected for evaluation in the soil pot trial with onion. These strains showed better performance for onion seedling growth promotion. Experiments were conducted in pots (upper diameter 8.5 cm, lower diameter 6.1 cm, and height 15.1 cm) containing 0.5 kg air-dried and sieved (2-mm mesh) soil. Soil (EC: 1536 μS cm^–1^, pH: 4.8; soil texture: sandy loam; Total N: 0.134±0.001%; available P: 182.0±5.3 mg kg^–1^; exchangeable K: 153.0±3.5 mg kg^–1^) was collected from an onion farm in Taichung county. To inoculate bacteria on seeds, 5-mL cell suspensions from 3-days-old colonies were added on filter paper to Petri dishes. Each treatment was replicated four times. The treatments included the following: half-dose fertilizer control (1/2F), half-dose fertilizer (1/2F)+individual strain, 1/2F+mixture of the 5 strains, full-dose fertilizer control (1F), 1F+individual strain, and 1F+mixture of the 5 strains. Four replicates for each strain were used (Lai *et al.*, 2008). Fertilization followed the Fertilizer Application Manual (Agriculture and Food Agency, Council of Agriculture, Executive Yuan, Taiwan, 2005). N, P, and K were applied as urea, single superphosphate, and potassium chloride, respectively (Table 1). Surface-sterilized seeds were immersed in 5-mL cell suspensions (A_600_=0.1) from 3-days-old bacterial cells, incubated at 25°C for 7‍ ‍d, and then transplanted to pots with unsterilized soil (sieved and air-dried soil). A 5-mL amount of the cell suspension in sterile water was added to pots immediately after transplanting and 1‍ ‍week after transplanting. Plants were grown in a growth chamber for 7‍ ‍weeks with a 12-h photoperiod (light: 153–179 μmole photons s^–1^ m^–1^) at 25°C. Pots were watered regularly and uniformly (Lai *et al.*, 2008). At the end of the pot experiment, inoculated bacteria were subjected to a new isolation to confirm that the introduced strains in the plants were the same. The results were positive because the same colony morphologies and colors were observed from the first isolation of the bacteria.

### Vermiculite pot trial on selected bacterial strains in three different vegetable crops

We used eight bacterial strains with high IAA production abilities to test growth changes in pak choy and sweet pepper planted in vermiculite (size 2) pots. To simplify the growth environment, we used vermiculite and not the soil sampled from the onion field.

Before planting, vermiculite was autoclaved once every day for 2 consecutive days. Unless indicated, bacteria preparation and inoculation, seed treatments, and pot sizes were all described previously (soil pot trial). We used four replicates for each strain.

The treatments included a control with half fertilizer (1/2F) and 1/2F+each of the 8 strains. Seeds were directly sown in vermiculite pots, and grown in a greenhouse for 4‍ ‍weeks at National Chung Hsing University, Taiwan. A 5-mL amount of the cell suspension in sterile water was added to pots when sown and 1‍ ‍week after sowing. Pots were watered regularly and uniformly (Lai *et al.*, 2008). Experimental timing was between June 1, 2019 and June 28, 2019. Climate conditions during the experiment, based on the Central Weather Bureau, Taiwan, were average humidity 78%, average air temperature 28.0°C, and average sunshine duration 4.0 h d^–1^. Plant height and root length were measured after 4‍ ‍weeks.

### Statistical analysis

Data were analyzed using SPSS 22.0 (IBM) with a one-way ANOVA and post-hoc test with the LSD test. *P*<0.05 was considered to be significant.

## Results

### Screening and identification of potential PGPB strains

**Fig. 1.**
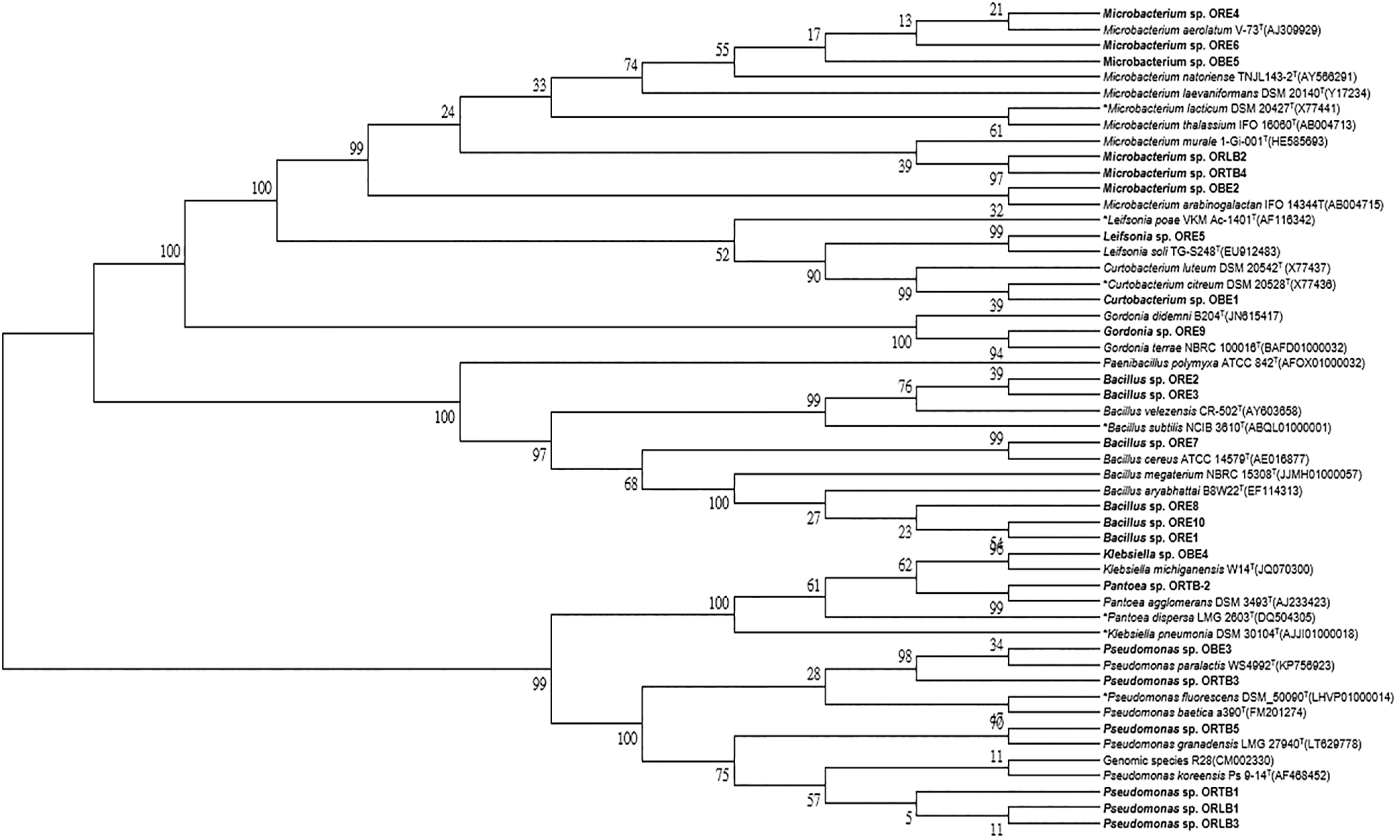
Phylogenetic tree of 23 bacteria (numbers at forks are confidence percentages from 1,000 replicates in the bootstrap analysis).

Twenty-three strains with PGP traits were isolated and identified from the bulbs, rhizosphere, and roots of onion plants (Table 2). The 16S rDNA sequences of all bacterial strains showed greater than 99% matching with the closest BLAST sequence, except that ORE4 was 96.3% identical with *Microbacterium hydrocarbonoxydans*, ORLB2 was 98.6% identical with *M. laevaniformans*, and ORTB2 was 97.6% identical with *Pantoea* sp.. BLAST results were further confirmed with a phylogenetic tree (Fig. 1). Five strains were isolated from bulbs, including *Curtobacterium* sp., *Klebsiella* sp., *Microbacterium* sp., and *Pseudomonas* sp.; 10 were from roots, including *Bacillus* sp., *Gordonia* sp., *Leifosonia* sp., and *Microbacterium* sp.; and 8 were from rhizosphere soil, including *Pantoea* sp., *Microbacterium* sp., and *Pseudomonas* sp.. The strains of *Bacillus* sp., *Microbacterium* sp., and *Pseudomonas* sp. were the dominant species isolated from onion (each included 6 out of the 23 strains isolated).

### Phosphate solubilization ability

**Fig. 2.**
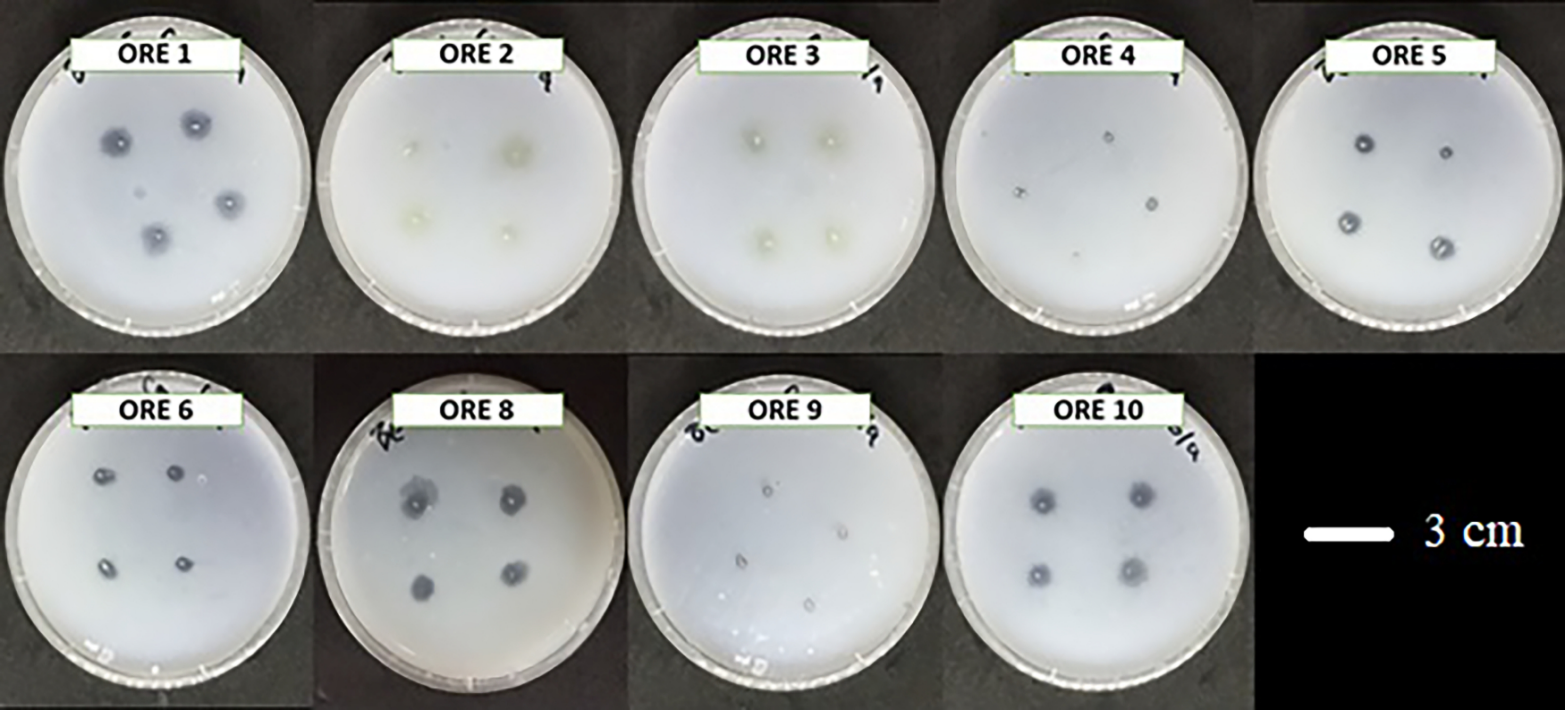
Nine bacterial strains isolated from onion roots with positive results for phosphorus solubilization.

**Fig. 3.**
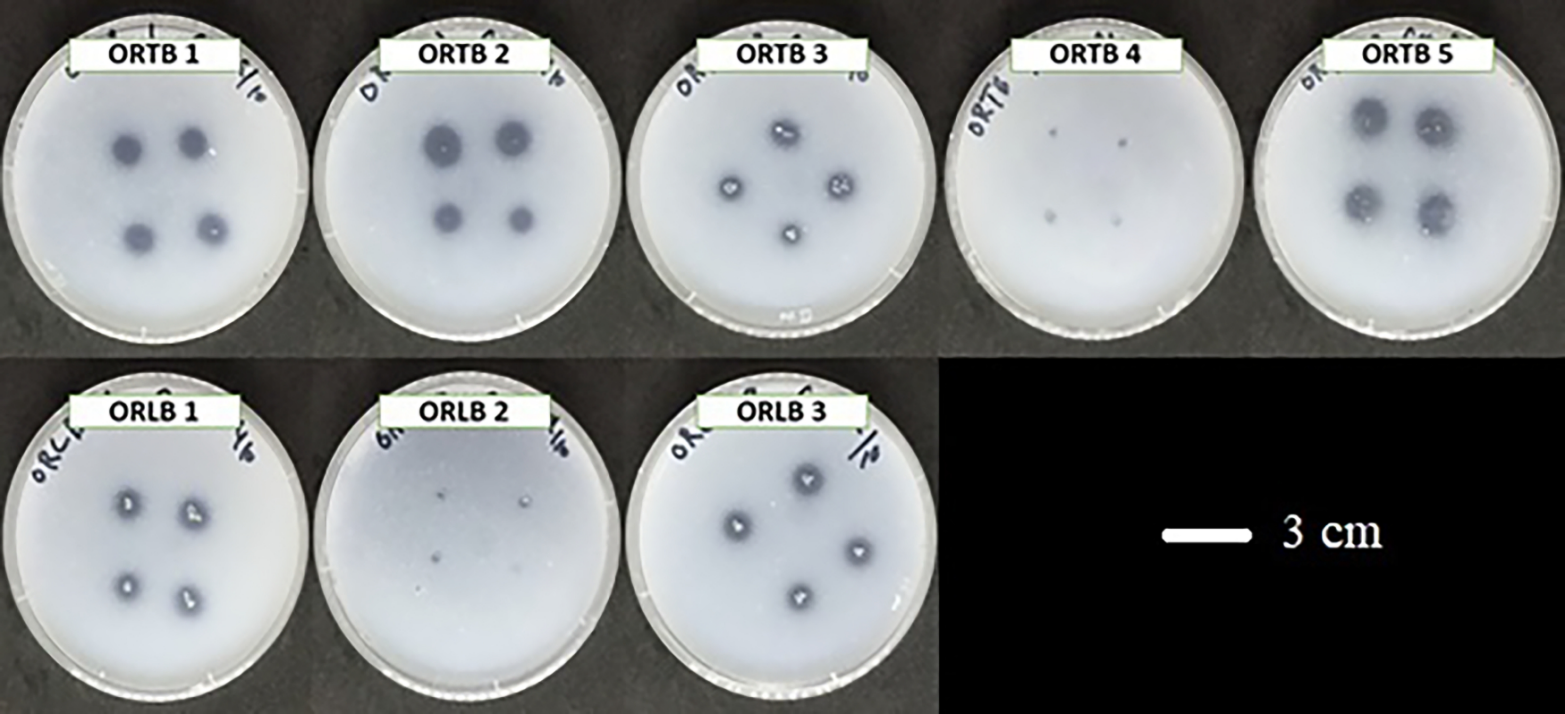
Eight bacterial strains isolated from tightly and loosely bound rhizospheres with positive results for phosphorus solubilization.

Twenty-two out of the 23 strains tested positive for phosphorous solubilization ability (Table 2); only *B. cereus* isolated from the roots showed negative results. After 7‍ ‍d, some strains were able to solubilize more phosphorus than other strains based on the size of the transparent circle (data not shown). ORE1, ORE5, ORE8, and ORE10 isolated from onion roots showed larger transparent circles than other strains (Fig. 2). ORTB1, ORTB2, ORTB3, ORTB5, ORLB1, and ORLB3 isolated from rhizospheric soil solubilized more phosphorus than other strains (Fig. 3). OBE1, OBE3, and OBE5 also solubilized more phosphate than other strains (data not shown).

### IAA production

As shown in Table 2, 15 (65.2%) of the strains produced IAA. However, 8 of these—ORE1, ORE5, ORE8, ORE10, ORLB2, ORTB2, ORTB3, and ORTB5—produced more IAA than the other seven. *B. megaterium* (ORE8) and *Pantoea* sp. (ORTB2) produced the largest amounts of IAA at 527 and 462 mg mL^–1^, respectively. The strains that produced the most IAA were isolated from the root endophyte (~103.8 mg mL^–1^ on average) and rhizosphere soil (~111.8‍ ‍mg‍ ‍mL^–1^ on average), but not from the bulb (~6.8‍ ‍mg‍ ‍mL^–1^ on average).

### ACC deaminase activity

All isolated strains were positive for ACC deaminase activity (Table 2). Some strains exhibited stronger ACC deaminase activity than the others: OBE2, ORLB2, ORTB2, ORTB3, ORTB4, and ORTB5 (>500 nmol α-ketobutyrate h^–1^ ΔOD_600_^–1^). The strains that produced the strongest ACC deaminase activity were isolated from the rhizosphere, with only one strain being isolated from the onion bulb (OBE2). The remaining strains exhibited between 97- and 500-nmol α-ketobutyrate h^–1^ ΔOD600^–1^ ACC deaminase activity.

### Nitrogen-fixing ability

All bacterial strains in semi-solid nitrogen-free medium were positive for acetylene reduction (or nitrogen-fixing) activity when analyzed by gas chromatography (Table 2). The range for all bacterial strains was between 2.9 and 25.9‍ ‍nmol C_2_H_4_ h^–1^, with OBE4 (25.9‍ ‍nmol C_2_H_4_ h^–1^) exhibiting the strongest nitrogenase activity and ORE7 (2.9 nmol C_2_H_4_ h^–1^) the weakest activity. Among the potential biofertilizers selected in the present study for subsequent plant experiments, ORTB3 had the highest nitrogen-fixing ability (8.1 nmol C_2_H_4_ h^–1^). Three out of the four bacterial strains with the highest abilities were from the onion bulb (~12.7‍ ‍nmol C_2_H_4_ h^–1^ on average). The strains with the lowest ability were from the root endophyte (4.9 nmol C_2_H_4_ h^–1^ on average), followed by those from the rhizosphere (6.0‍ ‍nmol C_2_H_4_ h^–1^).

### Petri dish trial on eight bacterial strains in onion, pak choy, and sweet pepper

We selected eight out of 23 strains for the Petri dish trial because of their high production levels of IAA (in bold in column 1, Table 2). Some selected strains, namely, ORE8 and ORTB2, significantly increased hypocotyl and radicle lengths simultaneously in onion, but decreased those in sweet pepper after 7‍ ‍d of growth (Table 3). These two strains also promoted onion growth the most. Hypocotyl lengths were 18.92, 35.62, and 36.74 mm in the onion control, ORE8, and ORTB2, respectively (showing an increase of 16.70 and 17.82 mm, respectively). However, ORE8 and ORTB2 significantly decreased the radicle length of sweet pepper; 24.34 mm in the control and 18.54 and 16.80 mm, respectively, in the strains (decreases of 5.80 and 7.54 mm, respectively).

The majority of strains caused a decrease in the hypocotyl length of pak choy, but an increase in radicle length. However, only ORE5 caused a significant increase in radicle length, while ORTB2 induced a significant decrease in hypocotyl length.

### Soil pot experiment for onions

**Fig. 4.**
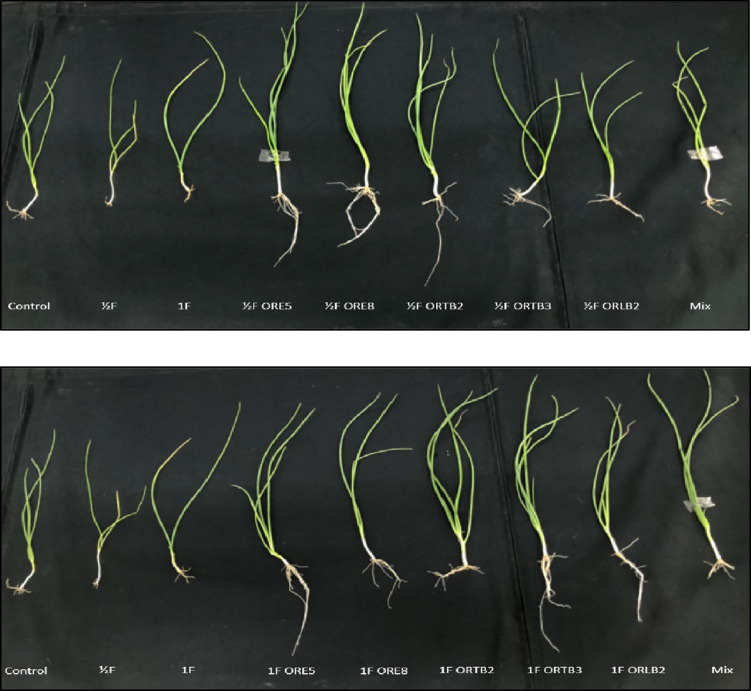
Onion plants inoculated with ORE5, ORE8, ORTB2, ORTB3, and ORLB2 grown with half-dose fertilizer (top) and full-dose fertilizer (bottom) in soil pots.

In the soil pot experiment, the majority of stains significantly promoted onion growth with half- and full-dose fertilizer (Table 4 and Fig. 4); these results were consistent with those obtained in the Petri dish trial. ORE5, ORE8, and ORTB2 grown in half-dose fertilizer had higher fresh and dry weights and longer shoot lengths than the half-dose fertilizer control and other treatments. With full-dose fertilizer, ORE5, ORTB2, and ORTB3 and the mixture of strains had higher fresh and dry weights and longer root lengths than the other treatments. High-dose fertilizer appeared to inhibit the performance of ORE5 and ORE8. However, ORTB2, ORTB3, and ORLB2 increased the growth of onion more with full-dose fertilizer. Furthermore, compare with the control treatment without fertilizer, the half- and full-dose fertilizer treatments all increased one leaf in the plant (data not shown).

### Vermiculite pot experiment

Similar results were obtained for pak choy in the Petri dish trial and vermiculite pot experiment. The majority of strains decreased shoot lengths, while all treatments increased root lengths (Table 5).

In sweet pepper, only shoot lengths decreased with ORTB2 and ORLB2, while shoot lengths increased with ORE1, ORE5, and ORE8. Increases in root lengths were observed with all treatments, particularly ORE5, ORE8, ORE10, ORTB5, and ORLB2 (Table 5).

## Discussion

### The identification of potential PGPB

In the present study, we isolated and identified 23 strains with PGP traits from the bulbs, rhizosphere, and endophytic roots of onion; the isolated strains included *Bacillus* sp., *Curtobacterium* sp., *Gordonia* sp., *Klebsiella* sp., *Leifsonia* sp., *Microbacterium* sp., *Pantoea* sp., and *Pseudomonas* sp. (Table 2). These species were previously reported to be PGPB (Apcarian *et al.*, 2006; Dastager *et al.*, 2009; Guiñazú *et al.*, 2013; Kang *et al.*, 2014; Bhutani *et al.*, 2018; Cedeño-García *et al.*, 2018; Gupta *et al.*, 2018; Yu *et al.*, 2018; Hmaeid *et al.*, 2019).

However, in addition to their plant and soil origins, *C. luteum* (OBE1), *G. terrae* (ORE9), and *K. oxytoca* (OBE4) have been isolated from human clinical specimens or human pathogens (Pham *et al.*, 2003; Funke *et al.*, 2005; Singh *et al.*, 2016). Therefore, this potential human pathogen origin restricts further agricultural applications of these bacteria. In the present study, we did not examine the effects of these bacteria on the growth of selected plants because of low IAA production levels. *B. megaterium* (ORE8) and *Pantoea* sp. (ORTB2) were selected because of their high IAA yield.

The six other bacterial strains selected for study were three *B. megaterium* strains isolated from root endophytes (ORE1, ORE8, and ORE10), *Leifsonia* sp. (ORE5) also isolated from root endophytes, and *M. laevaniformans* (ORLB2), *P. fluorescens* (ORTB3) and *P. moraviensis* (ORTB5) isolated from roots. *B. megaterium* and *P. moraviensis* were previously shown to produce a large amount of IAA (Bhutani *et al.*, 2018; Hassan and Bano, 2019). *M. laevaniformans* and *P. fluorescens* were also reported to be members of PGPB (David *et al.*, 2018; Tangapo *et al.*, 2018).

### Dual effects of IAA on different plants

In the present study, we selected eight bacterial strains as potential PGP candidates based mainly on their high IAA production abilities. IAA is the most common plant hormone that stimulates the growth and reproduction of plants (Taghavi *et al.*, 2010). It is the main auxin in plants and is involved in cell enlargement and division, tissue differentiation, and physiological processes. Approximately 80% of the rhizosphere has been proposed as a useful screening tool for the selection of endophytic- and rhizosphere-competent bacteria as rice growth-promoting agents (Etesami *et al.*, 2015). In the present study, 65.2% of bacterial strains had IAA production abilities.

Following rhizosphere colonization, some of these microorganisms penetrated the root tissue, thereby shifting their habitat from a rhizospheric to endophytic region (Gamalero and Glick, 2015); as shown in Table 1, most of the strains that produced larger amounts of IAA originated from both the roots and rhizosphere, but not from the bulb.

However, the large amounts of IAA produced by the strains does not always indicate the ability to promote plant growth. Persello‐Cartieaux *et al.* (2003) showed that *P. thivervalensis* at 10^5^ CFU mL^–1^ in Arabidopsis resulted in reproducible morphological changes, whereas inoculations with 10^6^ CFU mL^–1^ inhibited plant growth. In addition, *E. taylorae* inhibited plant growth by secreting a large amount of IAA (Sarwar and Kremer, 1995). Another *Enterobacter* strain was reported to suppress radish seedling growth by high IAA-producing species, and was regarded as a potent bio-herbicide candidate (Park *et al.*, 2015). Thus, IAA produced by bacteria interacts with plants in diverse manners from pathogenesis to phyto-stimulation, and the modulation of plant growth occurs within an optimal IAA concentration range. It is crucial to assess the IAA amount of potential PGPB before their application in the field.

Based on the results of our Petri dish trial (Table 3), ORE8 (*B. megaterium*) and ORTB2 (*Pantoea* sp.) inhibited the growth of sweet pepper, ORTB2 the growth of pak choy, and both strains the growth of onion, possibly because these two bacteria had the highest IAA production abilities among the bacteria tested (527 and 462 mg mL^–1^, respectively; the two bacterial strains also possessed similar nitrogen-fixing abilities (5.5 and 4.4 nmol C_2_H_4_ h^–1^) and phosphate-solubilizing abilities.

ORE8 and ORTB2 exerted different effects on the growth of onion, pak choy, and sweet pepper possibly because monocots (such as onion) perceive or respond differently to exogenous auxins than dicots due to the limited translocation or rapid degradation of exogenous auxins in monocots (Monaco *et al.*, 2002). Pak choy and sweet pepper were selected because they both have higher germination rates than weeds.

The results of the soil experiment on onion were consistent with those from the Petri dish trial. ORE8 and ORTB2 were among the half-dose fertilizer treatments that promoted onion growth the most, possibly because these strains produced a large amount of IAA.

However, in the vermiculate experiment, ORE8 and ORTB2 did not inhibit the growth of pak choy or sweet pepper; the growth of sweet pepper was promoted by ORE8. IAA may have leached from vermiculite, whereas in the Petri dish and soil, IAA remained in contact with the plant without being lost. Furthermore, the vermiculate experiment was conducted for 4‍ ‍weeks; therefore, IAA may have been diluted in the bigger plant or the plant and inoculated bacteria may have adapted such that growth was promoted after a period of time. Similar findings were reported by Kifle and Laing (2016): bacterial strains started to show a significant increase in growth after 30‍ ‍d of plant establishment. However, further studies are needed to verify this suggestion.

### Effects of ACC deaminase activity on plant growth

ACC deaminase, an enzyme that reduces ethylene levels in plants, is recognized as a PGP trait (Etesami *et al.*, 2014). Ethylene has also been reported to promote plant growth, but typically at very low concentrations (less than 0.1‍ ‍μL‍ ‍L^–1^) (Pierik *et al.*, 2006; Iqbal *et al.*, 2017). Penrose and Glick (2003) reported that more than 20 nmol α-ketobutyrate mg protein^–1^ h^–1^ of ACC deaminase activity was sufficient to permit a bacterium to grow on ACC and function as a PGPB. High activity levels of ACC deaminase (300 to 400 nmol α-ketobutyrate mg protein^–1^ h^–1^) for bacteria do not necessarily promote root elongation to any greater extent. However, the possibility of a dual effect from high ACC deaminase activity has not yet been reported.

In the present study, ACC deaminase activity levels were higher for ORLB2 (*M. laevaniformans*) and ORTB2 (*Pantoea* sp.) at 975.0 and 909.8 nmol h^–1^ ΔOD600^–1^, respectively. However, we did not observe the similar stimulation of pak choy seedling growth by the two stains (Table 3); exogenous ethylene was previously reported to inhibit pak choy growth (Bufler, 2009). The present results on ACC deaminase activity are consistent with previous findings reported by Penrose and Glick (2003).

### Nitrogen fixation

A previous study reported that the average nitrogenase activity of five bacterial strains isolated from sweet pepper was 3.1 nmol C_2_H_4_ h^–1^ (Terakado-Tonooka *et al.*, 2013). However, the average for the 23 strains in the present study was 7.0 nmol C_2_H_4_ h^–1^, with bacteria isolated from onion bulbs showing higher nitrogen-fixing abilities.

Among potential biofertilizers, ORTB3 (*P. fluorescens*) had the highest nitrogen-fixing ability (8.1 nmol C_2_H_4_ h^–1^). However, in the Petri dish trial (Table 3), ORTB3 significant increased onion hypocotyl lengths, but also inhibited sweet pepper growth. In the soil pot trial (Table 4), ORTB3 significantly promoted onion growth, particularly when full-dose fertilizer was applied. Nitrogen fixation by inoculating PGPB in rice was previously described (Keyeo *et al.*, 2011). Hence, the increase in growth by ORTB3 may be due in part to nitrogen-fixing ability in addition to other PGP traits.

### Potential PGPB for onion

In the Petri dish trial and soil pot experiment, we verified the stimulatory effects of the selected PGPB on onion growth. In the soil pot experiment, ORE5 (*Leifsonia* sp.), ORE8 (*B. megaterium*), and ORTB2 (*Pantoea* sp.) promoted onion growth the most with half-dose fertilizer, while similar results were obtained for ORE5 (*Leifsonia* sp.), ORTB2 (*Pantoea* sp.), and ORTB3 (*P. fluorescens*) with full-dose fertilizer.

We expected bacteria producing large amounts of IAA to inhibit the growth of dicot plants while enhancing the growth of a monocot plant, onion, in order to obtain selective PGPB with bioherbicidal potential. However, we obtained a negative effect in the Petri dish trial on other plants. but not in the vermiculite experiment, which showed that our potential PGPB to some extent also increased the growth of dicotyledon plants, such as pak choy and sweet pepper.

In conclusion, we isolated 23 bacterial strains from an onion plant. Most of the bacterial strains were *Microbacterium* sp., *Pseudomonas* sp., and *Bacillus* sp., which are well-known PGPB. In the present study, all strains possessed one or more PGP trait(s) (phosphorus solubilization, IAA production, ACC deaminase activity, and nitrogen fixation). Strains ORE5 (*Leifsonia* sp.), ORE8 (*B. megaterium*), and ORTB2 (*Pantoea* sp.) induced the greatest increases in onion growth during the vegetative stage with the application of half-dose chemical fertilizer. Furthermore, ORE8 (*B. megaterium*) and ORTB2 (*Pantoea* sp.) were the most promising biofertilizers for onion, but may simultaneously inhibit the seedling growth of other plants. Nevertheless, future field studies for the above mentioned bacteria need to be conducted in order to verify the effect on onion and the targeted plant species.

## Citation

Samayoa, B. E., Shen, F.-T., Lai, W.-A., and Chen, W.-C. (2020) Screening and Assessment of Potential Plant Growth-promoting Bacteria Associated with *Allium cepa* Linn.. *Microbes Environ ***35**: ME19147.

https://doi.org/10.1264/jsme2.ME19147

## Figures and Tables

**Table 1.** Fertilizers applied to crops.

	1^st^ Application^a^	2^nd^ Application	3^rd^ Application	4^th^ Application
	--------------------------------- g pot^–1^ ---------------------------------			
**Fertilization for onion**				
Urea	0.0825	0.033	0.033	0.017
Single superphosphate	0.295	0.126	0.000	0.000
KCl	0.051	0.025	0.025	0.000
**Fertilization for pak choy**				
Urea	0.202	0.100	0.091	0.010
Single superphosphate	0.403	0.388	0.111	0.111
KCl	0.103	0.103	0.000	0.000
**Fertilization for sweet pepper**				
Urea	0.200	0.190	0.190	0.099
Single superphosphate	0.500	0.450	0.450	0.009
KCl	0.609	0.609	0.555	0.555

^a^ Half of the quantities in each application were used for the treatments with half-dose fertilizer.

**Table 2.** Results of the BLAST search, phosphorous solubilization, indole acetic acid (IAA) production, 1-aminocyclopropane-1-carboxylate (ACC) deaminase, and nitrogen-fixation testing for 23 strains isolated from onion.

**Strain**	**Origin**	**BLAST Result**	**Phosphorus ** **solubilization**	**IAA production** (mg mL^–1^)	**ACC deaminase** (nmol h^–1^ ΔOD600^–1^)	**Nitrogen fixation** (nmol C_2_H_4_ h^–1^)	**Similarity**
OBE 1	Bulb	*Curtobacterium luteum*	++^b^	7.8	331.9	12.2	99.8%
OBE 2	Bulb	*Microbacterium esteraromaticum*	+	ND	636.9	15.1	99.5%
OBE 3	Bulb	*Pseudomonas gessardii*	++	5.3	311.6	4.3	99.9%
OBE 4	Bulb	*Klebsiella oxytoca*	+	ND	175.8	25.9	99.7%
OBE 5	Bulb	*Microbacterium hydrocarbonoxydans*	++	21	248.0	5.8	99.7%
**ORE 1**^a^	Root endophyte	*Bacillus megaterium*	++	202	294.4	3.4	99.7%
ORE 2	Root endophyte	*Bacillus amyloliquefaciens*	+	3.7	453.3	3.2	99.7%
ORE 3	Root endophyte	*Bacillus amyloliquefaciens*	+	4.3	140.0	3.5	99.7%
ORE 4	Root endophyte	*Microbacterium hydrocarbonoxydans*	+	ND	139.9	10.1	96.3%
**ORE 5**	Root endophyte	*Leifsonia sp.*	++	123	438.4	6.8	99.7%
ORE 6	Root endophyte	*Microbacterium hydrocarbonoxydans*	++	5.1	175.5	3.3	99.9%
ORE 7	Root endophyte	*Bacillus cereus*	–	ND	376.9	2.9	99.9%
**ORE 8**	Root endophyte	*Bacillus megaterium*	++	527	98.0	5.5	99.5%
ORE 9	Root endophyte	*Gordonia terrae*	+	ND	97.7	3.4	99.7%
**ORE 10**	Root endophyte	*Bacillus megaterium*	++	173	124.8	7.3	99.6%
ORLB 1	Rhizosphere	*Pseudomonas koreensis*	++	ND	471.9	7.1	99.6%
**ORLB 2**	Rhizosphere	*Microbacterium laevaniformans*	+	72	975.0	3.3	98.6%
ORLB 3	Rhizosphere	*Pseudomonas koreensis*	++	ND	345.7	7.3	99.5%
ORTB 1	Rhizosphere	*Pseudomonas koreensis*	++	16.3	419.8	6.9	99.7%
**ORTB 2**	Rhizosphere	*Pantoea *sp.	++	462	909.8	4.4	97.6%
**ORTB 3**	Rhizosphere	*Pseudomonas fluorescens*	++	235	649.3	8.1	99.8%
ORTB 4	Rhizosphere	*Microbacterium sp.*	+	ND	948.3	3.4	99.6%
**ORTB 5**	Rhizosphere	*Pseudomonas moraviensis*	++	109	531.1	7.1	99.2%

^a^ The strains marked in bold were selected in the pot test. ^b^ + positive with a smaller clear zone; ++ positive with a larger clear zone; – negative. ND, not detected.

**Table 3.** Growth of roots and shoots of onion, pak choy, and sweet pepper inoculated with potential plant growth-promoting bacteria for 7 d.

**Strain**	**Onion**			**Pak choy**			**Sweet pepper**	
	**Hypocotyl**	**Radicle**		**Hypocotyl**	**Radicle**		**Hypocotyl**	**Radicle**
	------------------------------------- mm -------------------------------------							
Control	18.92	7.50		12.52	8.62		12.94	24.34
ORE1	30.14*	5.40		12.26	9.24		13.14	22.60
ORE5	30.74*	12.26*		13.04	16.22**		11.96	20.94**
ORE8	35.62***	17.86***		12.34	12.16		11.20*	18.54***
ORE10	27.06	12.04*		10.56	11.44		12.14	21.26**
ORTB2	36.74***	16.26***		8.52**	8.32		10.46**	16.80***
ORTB3	34.78***	5.78		11.62	12.18		10.50**	17.36***
ORTB5	33.54**	9.02		11.62	13.70		11.72	14.16***
ORLB2	23.80	8.22		11.34	8.62		10.30**	16.66***

* *P*<0.05, ** *P*<0.01, *** *P*<0.001

**Table 4.** Fresh and dry weights and root and shoot lengths of onion grown in two batches in soil pots for 7‍ ‍weeks. Half-dose fertilizer (1/2F) was applied in batch 1, and full-dose fertilizer (1F) in batch 2.

**Batch 1**	**Fresh weight (g)**	**Dry weight (g)**	**Root length (mm)**	**Shoot length (mm)**	**Batch 2**	**Fresh weight (g)**	**Dry weight (g)**	**Root length (mm)**	**Shoot length (mm)**
1/2F	0.7	0.1	18	96	1F	1	0.1	27	124
ORE5	5.0***	0.2**	132**	228***	ORE5	4.4**	0.3**	127**	153*
ORE8	5.1***	0.3**	130**	215***	ORE8	2.9*	0.2	80*	140
ORTB2	5.1***	0.3**	125**	187***	ORTB2	6.8**	0.3**	150**	233***
ORTB3	2.2**	0.1	127**	181***	ORTB3	4.8**	0.3**	150**	230***
ORLB2	0.8	0.1	55	157**	ORLB2	3.8*	0.2	110**	207***
Mix	2.0**	0.1	55	109	Mix	4.7**	0.3**	82*	235***

* *P*<0.05, ** *P*<0.01, *** *P*<0.001

**Table 5.** Growth of roots and shoots of sweet pepper and pak choy in vermiculite pots for 4‍ ‍weeks.

**Strain**	**Pak Choy**			**Sweet Pepper**	
	**Shoot**	**Root**		**Shoot**	**Root**
	----------------------- mm -----------------------				
1/2F	90	82		75	100
ORE1	77	100		92*	122
ORE5	90	120*		92*	140**
ORE8	82	102		95*	160***
ORE10	80	115*		80	135*
ORTB2	87	92		72	120
ORTB3	90	105		75	115
ORTB5	82	115*		80	145**
ORLB2	80	92		72	137*

* *P*<0.05, ** *P*<0.01, *** *P*<0.001
