# Screening and Assessment of Potential Plant Growth-promoting Bacteria Associated with *Allium cepa* Linn.

**DOI:** 10.1264/jsme2.ME19147e

**Published:** 2020-03-20

**Authors:** Brian Estuardo Samayoa, Fo-Ting Shen, Wei-An Lai, Wen-Ching Chen

**Affiliations:** 1 International Master Program of Agriculture, National Chung-Hsing University, Taichung, Taiwan 40227; 2 Department of Soil and Environmental Science, National Chung-Hsing University, Taichung, Taiwan 40227; 3 Innovation and Development Center of Sustainable Agriculture (IDCSA), National Chung Hsing University, Taichung, Taiwan 40227; 4 International Bachelor Program of Agribusiness, National Chung-Hsing University, Taichung, Taiwan 40227

Vol. 35, No. 2, 2020 doi:10.1264/jsme2.ME19147

 

p. 1, left column

**Incorrect**

(Fatih, 2018)

 

**Correct**

(Hanci, 2018)

 

p. 9, right column

**Incorrect**

Fatih, H. (2018) A comprehensive overview of onion production: worldwide and Turkey. IOSR J Agric Vet Sci 11:17-27.

 

**Correct**

Hanci, F. (2018) A comprehensive overview of onion production: worldwide and Turkey. IOSR J Agric Vet Sci 11:17-27.

